# Homemade laparoscopic simulator^1^

**DOI:** 10.1590/s0102-865020190100000006

**Published:** 2019-12-09

**Authors:** Thiago da Costa Travassos, Edison Daniel Schneider-Monteiro, André Meirelles dos Santos, Leonardo Oliveira Reis

**Affiliations:** I MD, UroScience, Pontifícia Universidade Católica de Campinas (PUC-Campinas), Sao Paulo, Brazil. Conception and design of the study, technical procedures, analysis and interpretation of data, manuscript writing; II PhD, Physician at Hospital, PUC-Campinas, Sao Paulo, Brazil. Interpretation of data, manuscript preparation; III Full Professor, UroScience, Department of Urology, School of Medical Sciences, PUC-Campinas, Sao Paulo, Brazil. Conception and design of the study, technical procedures, analysis and interpretation of data, manuscript writing

**Keywords:** Laparoscopy, Simulation Training, Teaching

## Abstract

**Purpose:**

To describe a guide for the construction of a laparoscopic training simulator.

**Methods:**

Step-by-step description of an inexpensive and easy to assemble homemade laparoscopic training box, capable of simulating the laparoscopic environment in its peculiarities to enable technical skills training.

**Results:**

The total cost of the materials for the construction of the simulator was US$ 75.00 (about R$ 250.00 “reais”) and it can be reduced to US$ 60.00 if the builder judges that there is no need for internal lighting. The use of real trocars imposes the same challenges as real surgeries regarding positioning, visibility and limitation of movements.

**Conclusion:**

The proposed economical and efficient alternative can contribute to the teaching and practice of laparoscopic surgical technique worldwide, benefiting surgeons and patients.

## Introduction

The popularization of laparoscopic surgery since its introduction in the 1980s has brought with it the need for surgeons to adapt to this new surgical modality and to acquire the necessary skills to master this technique.

Anatomical knowledge as well as manual dexterity are essentials for laparoscopy, whose main challenges lay on ambidextrous, loss of tactile sensitivity and bi-dimensional images with loss of depth. The fact is that only performing actual laparoscopic operations the surgeon can become truly competent; however, patient safety issues, costs, time constraints and logistics, inevitably limit the training opportunities for beginners in the operating room^1^ ^–^ ^7^.

For decades, surgical training was based on the famous “see, do, teach” model credited to William Halsted in 1904. Today, with the evolution of minimally invasive surgeries, basic laparoscopic educators know that surgical skills need to be taught outside the operating theater and most training uses a variety of models, such as black boxes, virtual simulators, animals, and corpses^1^ ^,^ ^2^.

It is known that standardization, easy reproducibility of exercises, immediate feedback after each training, with acceptable cost provides benefits on coaching in laparoscopy, leading to the easy transference of techniques acquired to real surgeries, culminating in a shorter learning curve and surgeons and patients benefits^2^ ^,^ ^3^.

In the pursuit of this objective, we have developed at UroScience lab a step-by-step guide for the construction of a laparoscopic training box, which is inexpensive, easy to assemble and capable of simulating the laparoscopic environment in its peculiarities to enable the training of novice surgeons and experienced laparoscopists.

## Methods

### The box

**Figure 1:**
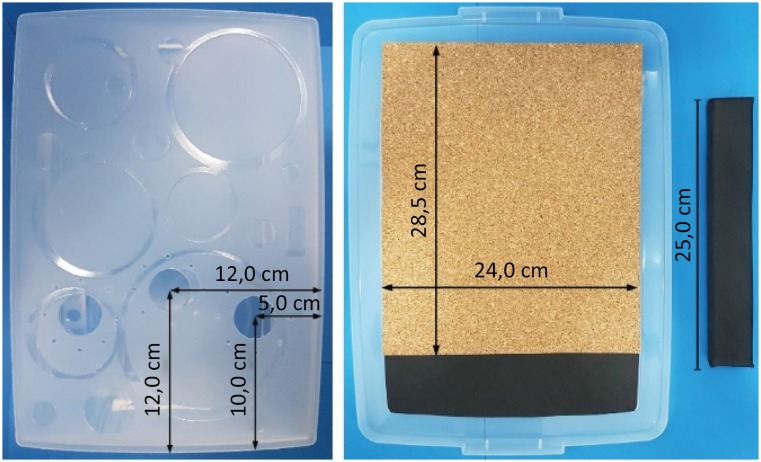
Location of the trocars’ holes at the bottom of the simulator. Position of the corkboard on the lid and camera holder appearance.

**Figure 2:**
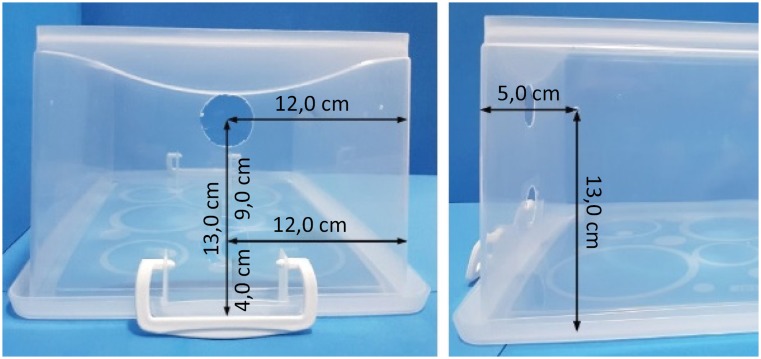
Holes made in one of the small sides of the box to pass the wires of the camera and the luminaire. 3 mm holes made in each of the bigger sides of the simulator to fix the camera holder.

We use a transparent plastic box of 15 liters (29.7 cm × 44.0 cm × 19.0 cm), (Order^®^, bel line). It is important to choose a box that has some resistance to making holes without cracking its structure. Three holes were made with a 1.1 / 4’ (32 mm) hole saw at the bottom of the box, due to the higher strength of the bottom compared to the cap. It is important that an expansion is made to adapt the drill bit of the hole saw avoiding cracks in the box structure. We used sequentially drill bits of 3 mm, 5 mm and 6 mm and only then the holes were made with the hole saw. These holes will be used for the installation of the trocars. Around each of these holes another 8 holes (in 2 groups of 4 holes, in “x” arrangement) of 3 mm were made for later fixing, with cotton threads, the EVA (Ethylene Vinyl Acetate) sheets. Two more holes (32 mm with hole saw) were made in one of the smaller sides of the box, following the same principles as the others; these holes will be used to install the camera and the light source. On each of the larger sides a 3 mm hole was made for later fixing the camera holder. The locations and distances of each of the orifices are shown in Figures 1 and 2.

### External structure

**Figure 3:**
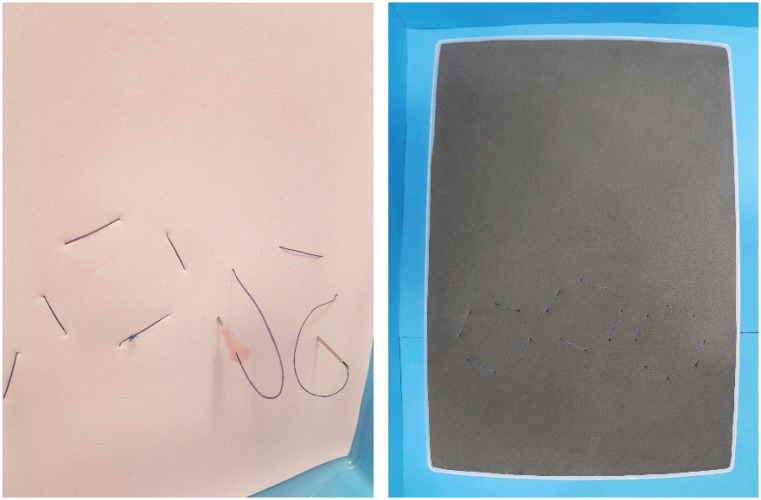
Layers of EVA fixed with 2-0 cotton threads, by a 40.0 × 1.2 mm (18 G × 1 1/2”) needle. Fixation through the 3 mm holes made around of the 32 mm holes at the bottom of the simulator.

Three layers of EVA (original sheet 40 × 60 cm, with a thickness of 2 mm) were glued, with plastic glue, in the side of the trocar's holes, one external and two internal. The box itself was used to demarcate the shape and size of the pieces of the original sheet. The first (outer) must be glued in isolation from the others to allow the fixation through the 3 mm holes, using 2-0 cotton threads, by a 40.0 × 1.2 mm (18 G × 1 1/2″) needle (Fig. 3). The other two can be glued simultaneously and fixed in the same way. Another sheet of EVA was glued onto the outside of the box, with aesthetic purpose.

### Internal structure

In the inside of the lid a corkboard of 24.0 × 28.5 cm was glued together with a small piece of EVA to finish the lid, as shown in Figure 1. For the camera holder we used a 25.0 × 2.0 × 3.0 cm piece of wood, coated with EVA. The holder was fixed to the box frame with a screw of 4.2 × 32 mm, on each side, passed through a shelf fastener (with aesthetic purpose).

### Camera and lighting

A Logitech^®^ camera, model c270 (HD 720p), attached to the camera holder with cable tie, was used. The chosen light source was a NSBAO^®^ (Bigspace, model YG-5933), flexible table lamp, with 14 LEDs (Light Emitting Diode), 3 levels of intensity and maximum brightness of 90 lumens. The base of the luminaire was glued with double-face adhesive tape, reinforced with cyanoacrylate glue (Super Bonder^®^), on the small side of the box. The flexible stem was passed behind the camera holder with the LEDs pointing toward the center of the box. The stem was attached to the top of the box with a rubberized cable organizer. The camera and luminaire wires were passed separately through the previously made holes in the smaller side.

### Finishing

**Figure 4:**
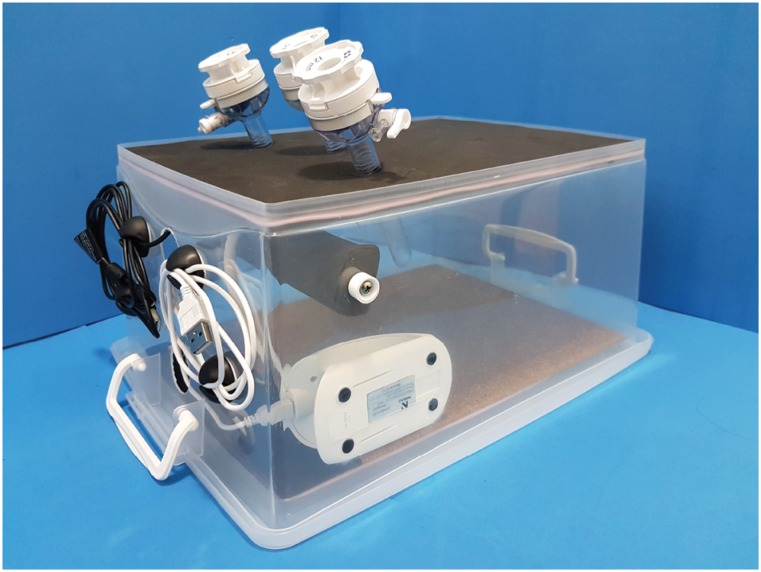
Simulator final aspect: camera attached to the camera holder with cable tie. Flexible table lamp passed behind the camera holder with the LEDs pointing toward the center of the simulator.

Three trocars were installed through linear cuts with scalpel blade, in the EVA sheets, in the topography of each of the holes of the box, to facilitate the entry of the trocars. Four rubber organizers were glued on the outside of the smaller face of the box to organize the camera and luminaire wires. The final appearance is shown in Figure 4.

## Results

The total cost of the materials for the construction of the box was US$ 75.00 (about R$ 250.00 “reais”) and it can be reduced to US$ 60.00 if the builder judges that there is no need for internal lighting.

The advantages of our box, besides its low cost, lightness and versatility, are the possibility to work only with the ambient light which further decreases the cost of the box, possibility of recording for analysis of movements after training and possibility to observe the movements performed by the surgeon, from an outside, three dimension, perspective.

The use of real trocars imposes the same challenges as real surgeries regarding positioning, visibility and limitation of movements.

## Discussion

We believe that the possibility of access to more economical and equally efficient alternatives to the current “black boxes”, available in the market, can contribute to the teaching and practice of laparoscopic surgical technique worldwide.

From the 80's, the art of surgery began an unprecedented process of transformation, with minimally invasive surgery assuming an important role in this scenario. Among its advantages we could mention less postoperative pain, shorter hospital stay, faster return to usual activities, better aesthetic result, significant reduction of overall costs, especially considering the period of retirement from work activities, and a lower rate of infections^1^ ^–^ ^5^.

In 2006, in the United States, only 55% of residency programs had facilities for laparoscopic training. In 2014, only 73% of the programs in all North America had teaching in laparoscopic skills^2^ ^,^ ^3^.

In Latin America, including Brazil, there is no established teaching and practicing model for training laparoscopic skills to be followed, nor validated tools for its evaluation. Nevertheless, studies have shown that those who practice laparoscopic skills in a simulated environment verify good acceptance and also improvement of their technical skills^4^.

## Conclusion

The present article presents the instructions to build a homemade laparoscopic training simulator, with low cost and easy assembly, with potential to improve technical skills in the environment of laparoscopy surgery, benefiting surgeons and patients.
